# 

**Published:** 1828-04-01

**Authors:** 


### CONTENTS

OF THE

### MEDICO-CHIRURGICAL REVIEW.

No. XVI. APRIL 1, 1828.

(*First Department.*)

### ANALYTICAL REVIEWS.

I. Eclectic Review on Pulmonic Inflammation .. .. .. .. .. .. .. 289
II. Dr. Cheyne on Malingering .. .. .. .. .. .. .. .. .. .. .. .. .. 309
III. Dr. Bright on Dropsical Effusion from Liver Disease .. .. .. .. .. .. .. 313
IV. Mr. Lawrence on Dislocations of the Vertebræ .. .. .. .. .. .. .. .. 320
V. Mr. Salmon on Stricture of the Rectum .. .. .. .. .. .. .. .. .. .. 325
VI. Medico-Chirurgical Transactions—Nævi Materni, &c. .. .. .. .. .. .. .. 333
VII. Dr. Abercrombie on the Brain and Spinal Cord .. .. .. .. .. .. .. .. 337
VIII. Dr. Cazenave on Rheumatism .. .. .. .. .. .. .. .. .. .. .. .. .. 351
IX. Medico-Chirurgical Transactions: Mr. Earle on Paraplegia, &c. .. .. .. .. .. 354
X. Dr. Horner on the Mucous Membranes .. .. .. .. .. .. .. .. .. .. .. 361
XI. Dr. Macculloch on the propagation of Malaria .. .. .. .. .. .. .. .. 370
XI. (*bis.*) On Medical Education .. .. .. .. .. .. .. .. .. .. .. .. .. 379
XII. Dr. Monro on Hydrocephalus .. .. .. .. .. .. .. .. .. .. .. .. .. 385
XIII. Mr. Wallace on a Fungous Eruption—Dr. Elliotson on Diarrhœa .. .. .. .. 401
XIV. Dr. Haslam on the Intellectual composition of Man .. .. .. .. .. .. .. 405
XV. Mr. Annesley on the Diseases of India .. .. .. .. .. .. .. .. .. .. .. 409
XVI. M. Koecker on Diseases of the Jaws .. .. .. .. .. .. .. .. .. .. .. 425
XVII. M. Laennec on Angina Pectoris .. .. .. .. .. .. .. .. .. .. .. .. 428

### PERISCOPE.

1. Division of Study and Practice in Medicine and Surgery .. .. .. .. .. .. 433
2. Practice of Medicine and Surgery .. .. .. .. .. .. .. .. .. .. .. .. .. 434
3. Medical Journals for the use of the Periscope .. .. .. .. .. .. .. .. .. 434
4. On a peculiar Species of Traumatic Delirium, by M. Dupuytren .. .. .. .. .. 435
5. Fractures of the Cervix Femoris, within the Capsule .. .. .. .. .. .. .. 437
6. Moral and Pathological Effects of Gambling .. .. .. .. .. .. .. .. .. .. 438
7. On Injuries of the Hip-Joint .. .. .. .. .. .. .. .. .. .. .. .. .. .. 438
8. On the Nature and Treatment of Traumatic Tetanus .. .. .. .. .. .. .. .. 439
9. Edinburgh Medical and Surgical Journal—its history .. .. .. .. .. .. .. 440
10. On the Treatment of Caries of Bone, by Dr. Nicol .. .. .. .. .. .. .. .. 441
11. London Medical Repository—its origin and success .. .. .. .. .. .. .. 443
12. Dr. Carter on Intermittent Fevers .. .. .. .. .. .. .. .. .. .. .. .. 443
13. London Medical Gazette—observations on this new Journal .. .. .. .. .. .. 444
14. Mr. Bell on the Diseases and Accidents of the Hip-Joint .. .. .. .. .. .. 444
15. Medical and Physical Journal—its origin and success .. .. .. .. .. .. .. 446
16. Mr. James on the Circulation .. .. .. .. .. .. .. .. .. .. .. .. .. .. 446
17. Sir G. Gibbes on Animalcula, Monades, Life, &c. .. .. .. .. .. .. .. .. 446
18. Intelligence from Constantinople—Secrets of the Seraglio .. .. .. .. .. .. 447
19. Dr. Thomas on Paraplegia .. .. .. .. .. .. .. .. .. .. .. .. .. .. .. 447

20. Dr. Elliotson on Chronic Affections of the Brain .. .. .. .. .. .. .. .. .. 448
21. Cooper *v.* Lawrence—or, the Trephine and the Scalpel .. .. .. .. .. .. 448
22. Duel between Dr. Forbes and Mr. Thompson .. .. .. .. .. .. .. .. 448
23. State of the Pupil in deep Intoxication. *(St. George's Hospital.)* .. .. .. .. 449
24. Strangulated Hernia. *(ib.)* .. .. .. .. .. .. .. .. .. .. .. .. 449
25. Anomalous Œdematous Swelling of the Leg—*(Middlesex Hospital.)* .. .. .. 450
26. Abscess between the Bladder and Pubes.—*(St. Thomas's.)* .. .. .. .. .. 451
27. Strangulated Hernia.—*(ib.)* .. .. .. .. .. .. .. .. .. .. .. .. 451
28. Fracture of the 4th and 5th Cervical Vertebræ.—*(Bartholomew's.)* .. .. .. 452
29. Morbid Growth in the Femur.—*(ib.)* .. .. .. .. .. .. .. .. .. .. 452
30. Hernia—adherent Epiplocele.—*(ib.)* .. .. .. .. .. .. .. .. .. .. 452
31. Laceration of the Urethra, &c.—*(Guy's.)* .. .. .. .. .. .. .. .. .. 453
32. Fatal Disease of the Brain, mistaken for Stomach Disorder .. .. .. .. .. 454
33. Thames Water .. .. .. .. .. .. .. .. .. .. .. .. .. .. .. .. 455
34. Remarkable case of Sympathetic Apoplexy .. .. .. .. .. .. .. .. .. 455
35. Liberty of the Press ; more Law-suits .. .. .. .. .. .. .. .. .. .. 456
36. An Essay on Delirium Tremens, by Dr. Coates .. .. .. .. .. .. .. .. 457
37. Hydatids of the Liver cured by an operation.—*(Hôtel Dieu, Paris.)* .. .. .. 459
38. M. Dupuytren on Stricture of the Lacrymal Duct.—*(ib.)* .. .. .. .. .. .. 460
39. On Amputation in Hospital, and on the Field of Battle.—*(ib.)* .. .. .. .. 461
40. M. Hellis on the Effects of Fear.—*(Hôtel Dieu, Rouen.)* .. .. .. .. .. 462
41. Hydro-pneumo-thorax—disease of the heart.—*(Hôpital Cochin.)* .. .. .. 462
42. Dr. Cazenave on Urticaria Intermittens.—*(Hôpital St. Louis.)* .. .. .. .. 464
43. Case of Fractured Ribs.—*(Royal Infirmary of Edinburgh.)* .. .. .. .. .. 465
44. Drs. Graves and Stokes on Cutaneous Diseases.—*(Meath Hospital.)* .. .. .. 465
45. History of Aneurism—Mr. Wardrop's operations .. .. .. .. .. .. .. .. 467
46. Dr. Lee on Arm and Shoulder Presentations .. .. .. .. .. .. .. .. .. 470
47. Mr. Rose on Purulent Depôts, after Wounds, &c. .. .. .. .. .. .. .. 470
48. Discussion on Auscultation and Percussion .. .. .. .. .. .. .. .. .. 470
49. Dr. Mott's Ligature of the Common Iliac Artery .. .. .. .. .. .. .. .. 472
50. Disease of the Heart—dissection.—*(Coldstream Guards' Hospital.)* .. .. .. 474
51. Bleeding in Erysipelas—false statements in the Lancet.—*(St. George's Hospital.)* 476
52. Popliteal Aneurism—flagrant mis-statements in the Lancet.—*(ib.)* .. .. .. 476
53. Secondary Hæmorrhage with cases.—*(Middlesex Hospital.)* .. .. .. .. .. 477
54. Fracture of the 7th Cervical Vertebra.—*(St. Thomas's Hospital.)* .. .. .. 478
55. Malignant bleeding Polypus.—*(Bartholomew's Hospital.)* .. .. .. .. .. 479
56. Strangulated Exomphalos, &c.—*(ib.)* .. .. .. .. .. .. .. .. .. .. 479
57. College of Physicians *v.* Dr. Harrison .. .. .. .. .. .. .. .. .. .. 480
58. Liberality and Illiberality .. .. .. .. .. .. .. .. .. .. .. .. .. 480
59. Calumnies of the Lancet against Messrs. Keate and Earle .. .. .. .. .. 480
60. Discussion on Thoracic Diseases in the Med.-Chir. Society .. .. .. .. .. 481
61. Foreign Bodies in Bodies Natural .. .. .. .. .. .. .. .. .. .. .. 483
62. Discussion on Delirium Tremens in the Westminster Society .. .. .. .. 484
63. Dr. Abercrombie on a peculiar Disease of the Pericranium .. .. .. .. .. 485
64. Observations on Coup de Soleil, by Mr. Mitchell .. .. .. .. .. .. .. 486
65. Remarkable Disease of the Lungs, by Dr. Seymour .. .. .. .. .. .. .. 487
66. On the Staining of Blood-vessels, by M. Laennec .. .. .. .. .. .. .. 488
67. Dr. Abercrombie on a peculiar Affection of the Cranium .. .. .. .. .. 489
68. Irish College of Physicians .. .. .. .. .. .. .. .. .. .. .. .. .. 490
69. Prize Essay, and discussion on Tubercles .. .. .. .. .. .. .. .. .. 490

70. Remarkable disease of the Mucous Membrane of the Bladder.—(*La Charité.*) .. 492
71. Peripneumony in Children.—(*Hôpital des Enfans.*) .. .. .. .. 493
72. Drs. Graves and Stokes on Hepatic diseases.—(*Meath Hospital.*) .. .. .. 494
73. Drs. Graves and Stokes on the Pathology of Fever.—(*ib.*) .. .. .. .. 495
74. Dr. Cullen on Bronchotomy.—(*Royal Infirmary Edinburgh.*) .. .. .. 496
75. Pulsating Tumours of the Scalp.
1. Dr. Maclachlan's cases, with commentaries.—(*Glasgow Infirmary.*) .. 497
2. Mr. Wardrop's case, with commentaries.—(*Panton Square.*) .. .. 499
76. Sciatica cured by Moxa.—(*Bartholomew's.*) .. .. .. .. .. 501
77. Hydrocele treated on Baron Larrey's plan.—(*Guy's.*) .. .. .. .. 501
78. Peculiar Affection of the Thigh.—(*St. George's.*) .. .. .. .. .. 502
79. Furious Delirium treated by Mercury.—(*St. Thomas's.*) .. .. .. .. 503
80. Duties of the Coroner .. .. .. .. .. .. .. .. .. .. 504
81. Drs. Graves and Stokes on Inflammation by contiguity .. .. .. .. 505
82. Encysted Dropsy of the Abdomen cured by an operation .. .. .. .. 505
83. Violent Abdominal Contusion .. .. .. .. .. .. .. .. .. 506
84. Dr Vilette on Hydrarthrosis, or Dropsy of the Knee-joint .. .. .. .. 506
85. M. Hufeland on Diseases of the Fœtus in Utero .. .. .. .. .. 507
86. Bony Tumour obstructing the Pylorus .. .. .. .. .. .. .. 508
87. Ascites cured by the introduction of Wine Vapour .. .. .. .. .. 508
88. Extraordinary case of Paruria Erratica .. .. .. .. .. .. .. 509
89. Dr. Clanny on the Pathology of the Blood in Typhus .. .. .. .. .. 510
90. Improved Mode of performing Tracheotomy .. .. .. .. .. .. 511
91. On Hæmatemesis and Melæna .. .. .. .. .. .. .. .. .. 512
92. Erysipelas treated by Lancet Punctures .. .. .. .. .. .. .. 514
93. Latest Curriculum of the Royal College of Surgeons .. .. .. .. .. 515
94. Gangrenous Erysipelas—amputation—death.—(*Royal Infirmary Edinburgh.*) .. 517
95. Mr. C. Bell on the question of Amputation.—(*Middlesex Hospital.*) .. .. 518
96. Dr. Marsh on Vapour-Bath in Tetanus.—(*Steevens' Hospital Dublin.*) .. .. 520
97. Injury of the Head—Trephining.—(*Bartholomew's.*) .. .. .. .. .. 521
98. Peculiar Affection of the Cranial Bones.—(*St. George's.*) .. .. .. .. 522
99. Mr. Amesbury on the non-union of fractured Bones.—(*St. Thomas's.*) .. .. 524
100. Mr. Lawrence's Letter to Dr. Johnson, with Dr. Johnson's reply .. .. .. 525
101. Prosecution of Mr. Stanley .. .. .. .. .. .. .. .. .. .. 527
102. Libel.—Macleod *versus* Wakley .. .. .. .. .. .. .. .. .. 528
103. Dr. Bouillaud on Disease of the Biliary Ducts.—(*Hôpital Cochin.*) .. .. 529
104. Ophthalmia Porriginosa.—(*Liverpool Ophthalmic Infirm.*) .. .. .. .. 531
105. Compound Fracture of the Thigh—Mortification.—(*St. George's.*) .. .. .. 533
106. M. Stultz on the Humoral Pathology.—(*Strasburgh Hospital.*) .. .. .. 534
107. M. M. Boyer and Delpech on Pilimiction.—(*Hôpital de Montpellier.*) .. .. 535
108. M. Bayle on Inflammation of the Medulla Spinalis.—(*La Charité.*) .. .. 535
109. Extirpation of a Sarcocele followed by Tetanus.—(*Hosp. General.*) .. .. 537
110. On the Treatment of Burns by Cotton.—(*Glasgow Infirmary.*) .. .. .. 538
111. Mr. Brodie's case of Popliteal Aneurism—falsehoods of the Lancet .. .. 541
112. Potash and Opium in Traumatic Tetanus .. .. .. .. .. .. .. 542
113. Pathology in the Dissecting Room .. .. .. .. .. .. .. .. 542
114. On the Extract of Valerian in large doses .. .. .. .. .. .. .. 544
115. Discussion on Diabetes Mellitus .. .. .. .. .. .. .. .. .. 545
116. Examination of the Curriculum of the College of Surgeons .. .. .. .. 545
117. External Iliac Artery tied by Mr. Brodie, with remarks .. .. .. .. 547

118. Wound of the Knee—Amputation .. .. .. .. .. .. .. .. .. .. .. 549
119. Lancet Ratiocination on Mr. Stanley's case .. .. .. .. .. .. .. .. .. .. 550
120. Ammoniacal Sulphate of Copper in Epilepsy .. .. .. .. .. .. .. .. .. .. 552
121. Muriate of Iron in Softening of the Stomach .. .. .. .. .. .. .. .. .. .. 552
122. Ligature Ultra Tumores—fatal hæmorrhage from Leech-bites .. .. .. .. .. 552
123. Mr. Auchincloss on Spinal Diseases .. .. .. .. .. .. .. .. .. .. .. .. 553
124. Mr. Plymsoll on Compound Fractures .. .. .. .. .. .. .. .. .. .. .. .. 554
125. On Erysipelas treated by Incisions.—(*Bartholomew's*) .. .. .. .. .. .. .. 555
126. Case of Axillary Aneurism.—(*Bartholomew's*) .. .. .. .. .. .. .. .. .. 556
127. Ligature of the External Iliac.—(*Nottingham Hospital.*) .. .. .. .. .. .. 557
128. Aortic Aneurisms.—(*Hospice General.*) .. .. .. .. .. .. .. .. .. .. .. 559
129. Remarkable Case of Hepatic Disease.—(*Milit. Hosp.*) .. .. .. .. .. .. .. 560
130. Royal College of Physicians .. .. .. .. .. .. .. .. .. .. .. .. .. .. 561
131. Dr. Kellie on Tubercles in various Structures .. .. .. .. .. .. .. .. .. 562
132. Popliteal Aneurism—Secondary Hæmorrhage .. .. .. .. .. .. .. .. .. 564
133. Mr. Davidson on Chloride of Lime .. .. .. .. .. .. .. .. .. .. .. .. .. 566
134. Westminster Discussions on Fever .. .. .. .. .. .. .. .. .. .. .. .. .. 567
135. Literary Justice as seen in the Lancet .. .. .. .. .. .. .. .. .. .. .. 569
136. Sir A. Carlisle on Erysipelas .. .. .. .. .. .. .. .. .. .. .. .. .. .. 569
137. Mr. Abernethy's Lectures .. .. .. .. .. .. .. .. .. .. .. .. .. .. 570
Mr. A. on Physic and Fever .. .. .. .. .. .. .. .. .. .. .. .. .. .. 571
138. Influence of the Stomach on the Brain .. .. .. .. .. .. .. .. .. .. .. 572
139. Mr. Abernethy on Asthma, with Criticisms .. .. .. .. .. .. .. .. .. .. 573
140. On the Diagnosis of Hernia .. .. .. .. .. .. .. .. .. .. .. .. .. .. 575
141. Ice to Aneurisms .. .. .. .. .. .. .. .. .. .. .. .. .. .. .. .. .. 576
List of Subscribers .. .. .. .. .. .. .. .. .. .. .. .. .. .. .. .. .. 576

